# A new insight to biomarkers related to resistance in survived-white spot syndrome virus challenged giant tiger shrimp, *Penaeus monodon*

**DOI:** 10.7717/peerj.8107

**Published:** 2019-12-20

**Authors:** Farhana Mohd Ghani, Subha Bhassu

**Affiliations:** 1 Department of Genetics & Molecular Biology, Institute of Biological Sciences, Faculty of Science, University of Malaya, Kuala Lumpur, Malaysia; 2 Centre for Research in Biotechnology for Agriculture (CEBAR), University of Malaya, Kuala Lumpur, Malaysia

**Keywords:** Novel discovery gene transcripts, Survived WSSV challenged shrimps, *P. monodon*, Transcriptomics

## Abstract

The emergence of diseases such as white spot disease has become a threat to *Penaeus monodon* cultivation. Although there have been a few studies utilizing RNA-Seq, the cellular processes of host-virus interaction in this species remain mostly anonymous. In the present study, *P. monodon* was challenged with WSSV by intramuscular injection and survived for 12 days. The effect of the host gene expression by WSSV infection in the haemocytes, hepatopancreas and muscle of *P. monodon* was studied using Illumina HiSeq 2000. The RNA-Seq of cDNA libraries was developed from surviving WSSV-challenged shrimp as well as from normal healthy shrimp as control. A comparison of the transcriptome data of the two groups showed 2,644 host genes to be significantly up-regulated and 2,194 genes significantly down-regulated as a result of the infection with WSSV. Among the differentially expressed genes, our study discovered HMGB, TNFSF and c-Jun in *P. monodon* as new potential candidate genes for further investigation for the development of potential disease resistance markers. Our study also provided significant data on the differential expression of genes in the survived WSSV infected *P. monodon* that will help to improve understanding of host-virus interactions in this species.

## Introduction

*Penaeus monodon*, commonly known as the giant tiger shrimp, is an important aquaculture species that has been farmed for food for more than a century in Asian countries (FAO, 2017). However, over the past century, various new diseases affecting shrimp have emerged as a result of intensive aquaculture, the increasing global movement of aquatic animals and their products, and various human-caused sources of stress to the aquatic ecosystem (Walker & Winton, 2010). Farmed penaeid shrimp are in general more susceptible to disease outbreaks than freshwater prawns as they are farmed much more intensively (Biju Sam Kamalam, Saravanan & Ajith Stalin, 2009). Disease outbreaks in *P. monodon* stocks have become a particular concern in recent years (FAO, 2017), leading to a decline in the production of the shrimp species and its replacement in many cases by *Penaeus vannamei*—a species which is easier and simpler to farm and which is less prone to disease problems.

White spot syndrome virus (WSSV), a member of the *Nimaviridae* family, is one of the eight viral pathogens causing notifiable diseases in marine shrimp (OIE, 2017), and is one of the most destructive pathogens of farmed shrimp. WSSV was first reported in June 1992 in cultured *Penaeus japonicus*, the kuruma shrimp, in the Fujian Province of China and nearby Taiwan (Zhan et al., 1998; Jiang, 2001). The virus can cause mass mortality (80–100%) in cultured giant tiger shrimp within 5–10 days of the first clinical signs appearing (Chou et al., 1995).

Various techniques have been applied to study shrimp immune responses to viral infections, including cDNA microarray technology (Aoki et al., 2011; Lu, Hsiao & Wu, 2011; Shekhar et al., 2015) and next generation sequencing (Clavero-Salas et al., 2007; Zeng et al., 2013; Chen et al., 2013). Few researches involving transcriptome study with WSSV infection in shrimp and prawn have been performed to understand the effect of WSSV infection to the host immune system. Those researches include transcriptome analysis on *P. vannamei* (Chen et al., 2013; Santos et al., 2018), Chinese shrimp (*Penaeus chinensis*) (Li et al., 2013), Kuruma shrimp (*Penaeus japonicus*) (Zhong et al., 2017) and Oriental river prawn (*Macrobrachium nipponense*) (Zhao et al., 2018). However, although a number of immune-related proteins in shrimp have been identified detailing the interactions between viruses and the host innate immune system using these two techniques, the interactions between WSSV and the host intracellular environment have received less attention. Research on WSSV infection has been complicated by the lack of well-annotated genomic resources for host species (Verbruggen et al., 2016). Furthermore, Yue & Wang (2017) claimed that no genome sequence had been reported in shrimp species although efforts are taken to sequence shrimp species, such as *P. vannamei* and *P. monodon*. White spot syndrome virus is also known to infect most shrimp tissues and organs (Pradeep et al., 2012), including both immune-related and non-immune cells; and the expression of these different cell types to the pathogenesis of the virus is likely to be different (Leu et al., 2007). Hence, the present study focuses not only on immune-related tissues (hepatopancreas and haemocytes), but also looks into the host-virus interaction of non-immune-related tissue (muscle) and intracellular environment.

Large-scale and detailed assessments of the transcript abundance and transcript structure in host tissues can be obtained from the sequencing of the transcriptome (Grabherr et al., 2011). Thus, applying gene expression profiling to the interactions between the virus and shrimp can provide understandings into the mechanisms through which WSSV suppresses and destabilizes host defence responses (Chen et al., 2013). By providing a partial description of the transcribed regions in a target organism, transcriptome analysis can be a resource for identification and mining desirable gene traits (Leu et al., 2011). Specifically, by using the Illumina HiSeq 2000 Platform, the present study aims to provide valuable information on the differential gene expressions of *P. monodon* challenged with WSSV and to identify disease resistance genes for breeding purposes.

## Materials and Methods

### White spot syndrome virus propagation and preparation of virus inoculation

Juvenile *P. monodon* from local wild broodstock was used for the propagation of WSSV. These shrimp (15–20 g body weight), collected from a local commercial farm, were first tested by PCR to ensure that they were negative for WSSV (Kimura et al., 1996). The propagation of WSSV was achieved by feeding the juvenile shrimp minced WSSV infected muscle tissue in 40-L glass aquaria. During propagation, the salinity of the seawater was reduced drastically from 30 ppt to 15 ppt (at 28.0  ± 1.0 °C) to induce stress and ensure successful infection with 100% mortality. All the dead shrimp were then frozen to −80 °C and tested for WSSV using OIE primer pairs VP28 F (5′TACTCAGTCGACACCACCATGGATCTTTCTTTC′3) and VP28 R (5′TACTCACTGCAGTTACTCGGTCTCAGTGCCA3′) (Kimura et al., 1996).

White spot syndrome virus inoculation was prepared using the positively infected propagated shrimp, based on a method described by Supamattaya et al. (1998). Muscle tissues from the infected shrimp were homogenized and lysed in TN Buffer (20mM Tris.HCl, 0.4M NaCl; pH 7.4) and tissue homogenate was collected and centrifuged at 3,000 g for 10 min (4 °C). The supernatant was filtrated through a 0.20 µM sterile microfilter and the virus stock solution was stored at −80 °C until used. The WSSV copy numbers in the stock solution were determined by a method described by Mendoza-Cano & Sánchez-Paz (2013) using primer pairs VP28-140Fw (5′AGGTGTGGAACAACACATCAAG′3) and VP28-140Rv (5′TGCCAACTTCATCCTCATCA′3).

Crude viral extract prepared earlier was diluted serially to 10^−2^, 10^−4^, 10^−6^ and 10^−8^ in order to determine the lethal dose (LD_50_). Juvenile *P. monodon* (15–20 g body weight), obtained from Balik Pulau, Penang, Malaysia and checked by PCR to ensure they were negative for WSSV (Kimura et al., 1996), were maintained in 40-L glass aquaria (10 individuals per tank) containing seawater at a salinity of 30 ppt (at 28.0  ± 1.0 °C). An LD_50_ test was carried out based on Tassanakajon et al. (2006), by injecting 100 µL of each dilution factor of the virus into the ventral 3rd abdominal segment of the shrimp using a 1 mL syringe (29 G). Control animals were injected with 100 µL PBS. These LD_50_ experiments were carried out for seven days, during which the shrimp were monitored for activity and changes in behaviour. All shrimp were tested for WSSV using OIE primer pairs (Kimura et al., 1996).

### Challenge with WSSV

The shrimps used in the challenge test experiment originated from Mozambique, Africa, and were maintained on a commercial farm in Balik Pulau, Penang, Malaysia, for breeding purposes. Eighty juvenile shrimp from the F4 generation (age 60 days, 15–20 g body weight) were acclimatised for 1 week at the ambient temperature prior to the challenge test to allow them to recover from the transportation stress and were fed twice daily with commercial postlarval feed. The salinity of water was 30 ppt with a pH of 7.4–7.6. The water was renewed at a daily rate of 20%. All shrimp were checked as being WSSV negative by PCR (Kimura et al., 1996).

The experimental challenge test itself was performed using one 40-L negative control tank and seven 40-L WSSV-challenged tanks (10 individuals per tank). Artificial seawater (Forty Fathoms Marine Mix, Baltimore, MD) at 28  ± 1.0 °C and with a salinity of 30 ppt was used in the challenge test. The tanks were equipped with air diffusers to provide enough aeration as well as with water filters to maintain a clean and healthy environment. Each tank was covered with green netting to contain aerosol and minimize water temperature fluctuations.

All shrimp in the seven challenge tanks were injected with 100 µL of WSSV (4.11  × 10^5^ viral copies/µL) in their ventral 3rd abdominal segments using a 1 mL syringe (29 G). For the control group, the shrimp were injected with 100 µL PBS. All shrimp were fed with commercial shrimp pellets twice a day. The tanks were checked three times daily for moribund or dead animals. To reduce cannibalism in the experimental tanks, dead shrimp were removed daily after exposure to the virus throughout the test period. These dead shrimps were frozen immediately at −80 °C. The remaining WSSV-challenged shrimp were maintained in the tanks until mortality ceased at day 12 post challenge. The live shrimp at that point were counted as survivors. All the survivors were dissected, snapped frozen and stored at −80 °C, and subsequently tested for the presence of WSSV by PCR (Kimura et al., 1996) and qPCR (Mendoza-Cano & Sánchez-Paz, 2013).

### Generation of transcriptome data by next generation sequencing (Illumina HiSeq 2000)

Total RNA from hepatopancreas, haemolymph and muscle of the survived and control shrimp were isolated using an RNA Isolation Kit (Macherey’s-Nagel, Germany) according to the manufacturer’s protocol. The RNA was quantified by UV absorbance at 260 nm, and its quality was assessed by electrophoresis in 1% agarose gel. An equal amount of high-quality total RNA from each individual was then pooled for sequencing. A library construction and sequencing run were carried out by the Beijing Genome Institute (Hong Kong) on an Illumina HiSeq 2000 platform.

The raw sequencing reads were quality trimmed, and adaptor sequences were removed before assembly. The filtered high-quality sequences (cleaned reads) were *de novo* assembled using Trinity with default parameters (Grabherr et al., 2011). The overall assembly was produced by assembling the combined sequence data from both the surviving WSSV-challenged shrimp and the control samples. Both reads data sets for the different conditions were combined into a single target for Trinity assembly to generate a reference assembly that can be later use for analysing differential expression. The reads were separately aligned back to a single Trinity assembly for downstream analysis of differential expression. The reads data sets for the different conditions were combined for *de novo* assembly to avoid difficulty comparing the results across different conditions due to differences in assembled transcript lengths and contiguity. For functional annotation analysis, all the unigenes were compared with sequences in NCBI non-redundant (nr) protein and the Swiss-Prot, KEGG and COG databases using BLASTX programs (*E*-value < 0.00001) (Kanehisa et al., 2008). The genes were tentatively identified according to the best hits against known sequences. Functional annotation in gene ontology terms (GO) was produced using a BLAST2GO program (https://www.blast2go.com/) (Conesa et al., 2005).

In order to analyse the differential gene expression, the transcript levels were measured as FPKM (Fragments Per Kilobase of exon model per Million mapped reads) values to determine the relative abundance of each gene in the transcript (Mortazavi et al., 2008). FPKM were calculated by using the formula FPKM (A) = 10^6^C/(NL*10^−3^); where FPKM (A) is the expression of gene A, C equals to the number of reads that specifically aligned to gene A, N is the total number of reads that aligned to all genes, and L is the number of bases of gene A. After normalizing all unigenes to FPKM, differentially expressed genes between the survived over control samples were identified based on the significance of digital gene expression profiles developed by Audic & Claverie (1997). An FDR (false discovery rate) of 0.001 was used as the threshold of the *p*-value in the multiple tests to judge the significance of the difference in gene expression (Storey, 2002). Genes were considered differentially expressed in a given library when the *p*-value was less than 0.001 and a greater than two-fold change in expression between libraries was observed.

### Validation of NGS data & comparative transcriptome profiling analysis

To validate the Illumina HiSeq 2000 sequencing data, 10 immune-related genes with potential for disease resistance were chosen for quantitative RT-PCR analysis, using the same RNA samples as for the Illumina HiSeq 2000 sequencing (Table 1). First strand cDNA was synthesized from 1 µg of RNA using the ImProm-II™ Reverse Transcriptase (Promega). The qPCR reaction mixture consisted of 2X Power SYBR Green PCR Master Mix, each of the forward and reverse primers, and 1 µL of template cDNA. Primer sets were designed using the Primer-BLAST (http://www.ncbi.nlm.nih.gov/tools/primer-blast/) (Ye et al., 2012).

**Table 1:** Primers used for real-time RT-qPCR, showing nucleotide sequence and amplicon size.

Target gene	Primer	Primer sequence (5′–3′)	Amplification size
Heat shock protein 10	hsp10F	ACCTTCCCTGTGAGGACCTT	113
	hsp10R	TTTGTTCCCCTGTTCGACCG	
Heat shock protein 60	hsp60F	CAGTCCTGGCTCGCACTATT	97
	hsp60R	TCCACGGCCAACATAACTCC	
Heat shock protein90	hsp90F	GGAGACGCTCAACAAATGGC	182
	hsp90R	AGACTCTGCAAACCGTACCC	
Caspase	cascF	GCGAGCATCGTAGTCGAGTT	87
	cascR	GCACGAGGTTTTGTTCGCAT	
Carcinin like protein	carcF	ACATCGTAGCAGCACTTGGA	122
	carcR	GAAGTTCACGACGGCGACT	
Anti-lipopolysaccharide factor isoform 3	alf3F	CTACAAGGGGAGGATGTGGTG	85
	alf3R	CTTTCCAGCTACCCCGGAC	
Hemocyte homeostasis-associated protein	hhapF	TTTCCTTCGGTGGGTCATCG	78
	hhapR	AGTGCAAATCGTGCAACACC	
Crustacean hematopoietic factor	chfF	GTGCCCAATTTCTTCCACGTC	133
	chfR	GTGAAGGATGCACACCCGA	
Hepatopancreas kazal-type proteinase inhibitor 1A1	hepkpiF	ACTCTGGCAATTGGCTCGTT	81
	hepkpiR	GAGAACTACGACCCCGTGTG	
Kazal-type serine proteinase inhibitor 4	ksp14F	CGCCAGGCTAATACCTCCTC	74
	ksp14R	ACGGCGTGACCTACTCTAAC	

The expression levels of the selected genes were evaluated using the high-throughput microfluidic 192.24 BioMark™ HD Real Time PCR System (Fluidigm Corporation, CA, USA). The sample reaction mixtures were produced in a final volume of 5 µl containing 1.25 µl of preamplified cDNA (diluted 1:5), 2.5 µl of 2X TaqMan Gene Expression Master Mix (Applied Biosystems), 0.25 µl of 20X DNA Binding Dye Sample Loading Reagent (Fluidigm), 0.25 µl of 20X EvaGreen (Biotium) and 0.75 µl of 1X TE buffer. Primer reaction mixtures were produced in the same volume of 5 µl, containing 2.5 µl of 2X Assay Loading Reagent (Fluidigm), 1.25 µl of 20 µM of forward and reverse primer mix, and 1.25 µl of 1X TE buffer. Both sample and primer reaction mixtures were loaded into a dynamic array chip that was subsequently placed on the HX IFC controller (Fluidigm, South San Francisco, CA, USA) for loading and mixing. After approximately 50 min, the chip was transferred to the BioMark™ Real-Time PCR System (Fluidigm, South San Francisco, CA, USA).

The cycling program used consisted of 10 min at 95 °C, followed by 40 cycles of 95 °C for 15 s and 1 min at 60 °C. A melting curve analysis was performed after the RT-qPCR was completed, collecting fluorescence between 60 and −95 °C at 0.5 °C increments. The resulting data were analyzed using the BioMark™ Real-time PCR analysis software to obtain Ct values. The elongation factor (EF) gene was selected as an endogenous reference gene. The results were presented as changes in relative expression normalized to the arithmetic mean of the Ct values of the reference gene (Livak & Schmittgen, 2001). Statistical significance was determined by a one-way ANOVA, followed by Tukey’s test at *p* < 0.05.

### Molecular response of selected immune related genes in *P. monodon* triggered by WSSV infection

The experimental challenge consisted of one 40-L negative control tank and three 40-L WSSV-challenged tanks (10 individuals per tank). Challenge test followed method as mentioned in previous section. All shrimp of the three challenge tanks were injected with 100 µL of WSSV (2.7 × 10^−6^) into the ventral 3rd abdominal segment of the shrimp using a 1mL syringe (29 G). Control animals were injected with 100 µL PBS. Challenge test was terminated on day 12. Hepatopancreas, muscle tissue and haemolymph were collected from the WSSV challenged and control shrimp at 0, 3, 6, 12, 24, 36 and 48 h post infection (hpi) and 12 days post infection (dpi). Haemolymph was collected using a 1mL syringe (30 G needle) pre filled with 100 µL anticoagulant. The tissue was snapped frozen and stored at −80 °C. Total RNA from all tissue was extracted using TRIzol reagent (Qiagen) and first strand cDNA was synthesized from 1 µg of RNA using ImProm-II™ Reverse Transcriptase (Promega). RNA was quantified by UV absorbance at 260 nm and its quality was assessed by electrophoresis in 1% agarose gel. Primer sets were designed using the Primer-BLAST (http://www.ncbi.nlm.nih.gov/tools/primer-blast/) (Ye et al., 2012).

An additional experiment was set up to quantify the gene expression of the selected immune related genes by using an ABI 7500 Real-time Detection System (Applied Biosystems, Foster City, California, USA). Quantitative real-time PCR analysis (qRT-PCR) was carried out in a 20 µL reaction volume containing 50 ng of cDNA from each tissue, 2X of power SYBR Green Master Mix, 0.3 µM of each primer and 7.8 µl dH2O. The qRT-PCR cycle profile was 1 cycle of 95 °C for 10 s, followed by 40 cycles of 95 °C for 10 s, 60 °C for 20 s and 72 °C for 10 s. The data was analysed with the ABI 7500 SDS software (Applied Biosystems). The elongation factor (EF) gene was selected as endogenous reference genes. Statistical analysis was performed using GraphPad Prism 4 (GraphPad Software, Inc.). The results were presented as changes in relative expression normalized to the arithmetic mean of the Ct values of the reference gene (Livak & Schmittgen, 2001). Relative gene expression values were determined using the the 2^−ΔΔ*CQ*^ Livak method (Livak & Schmittgen, 2001). Statistical significance was determined by one-way ANOVA followed by Tukey’s test at *p* < 0.05 using GraphPad Software. All data was presented as relative mRNA expressed by means ± standard deviation.

### Sequence and statistical analysis of selected immune related genes in *P. monodon*

The nucleotide sequences were analyzed to search for open reading frame (ORF) by ORF Finder (NCBI) (https://www.ncbi.nlm.nih.gov/orffinder/). The ORF translated amino acid sequences were search against the NCBI non-redundant (nr) protein database using BLASTp program (*E*-value < 0.00001) (Kanehisa et al., 2008). The protein orthologs were identified according to the best hits against known sequences. Multiple sequence alignments of the protein orthologs were generated using the Clustal Omega (McWilliam et al., 2013).

The amino acids under selective pressure were detected by the ratio of the rate of non-synonymous substitutions (dN) to the rate of synonymous substitutions (dS) for each codon, calculated with Selecton web server (Stern et al., 2007), based on M8 evolutionary model which allows for positive selection. Additionally, the dS and dN variances: Var(dS) and Var(dN), were estimated respectively. With this information, dN/dS was calculated and the null hypothesis of no selection (H0: dN = dS) versus the positive selection hypothesis (HA: dN >dS) using the *Z*-test: *Z* = (dN −dS)/$\sqrt{(\mathrm{V ar}(\mathrm{dS})+\mathrm{V ar}(\mathrm{dN}))}$was tested. *Z* tests calculations were performed using the MEGA software (Tamura et al., 2013).

## Results

### Viral copies number and LD_50_

The WSSV stock solution contained 4.11 × 10^11^ viral copies/µL. For determination of LD_50_, all WSSV-injected shrimp showed no gross signs of typical white spot disease. However, mortality of the shrimp reached 100% by the fourth day post infection in shrimp injected with undiluted WSSV stock solution and WSSV solution with dilution factor 10^−2^ and 10^−4^. Nearly half of the shrimp injected with WSSV solution with dilution factor 10^−6^ died by the seventh day post infection. Shrimp injected with WSSV solution with dilution factor 10^−8^ appeared healthy and fed well until the end of LD_50_ test. WSSV stock solution with dilution factor 10^−6^ was chosen to use for challenge test (4.11 × 10^5^ viral copies/µL). According to Chen et al. (2013), a dose of about 1 ×10^5^ WSSV copies/g sufficient to cause 100% mortality in 5–7 days.

### Detection of WSSV in surviving giant tiger shrimp

**Figure 1:**
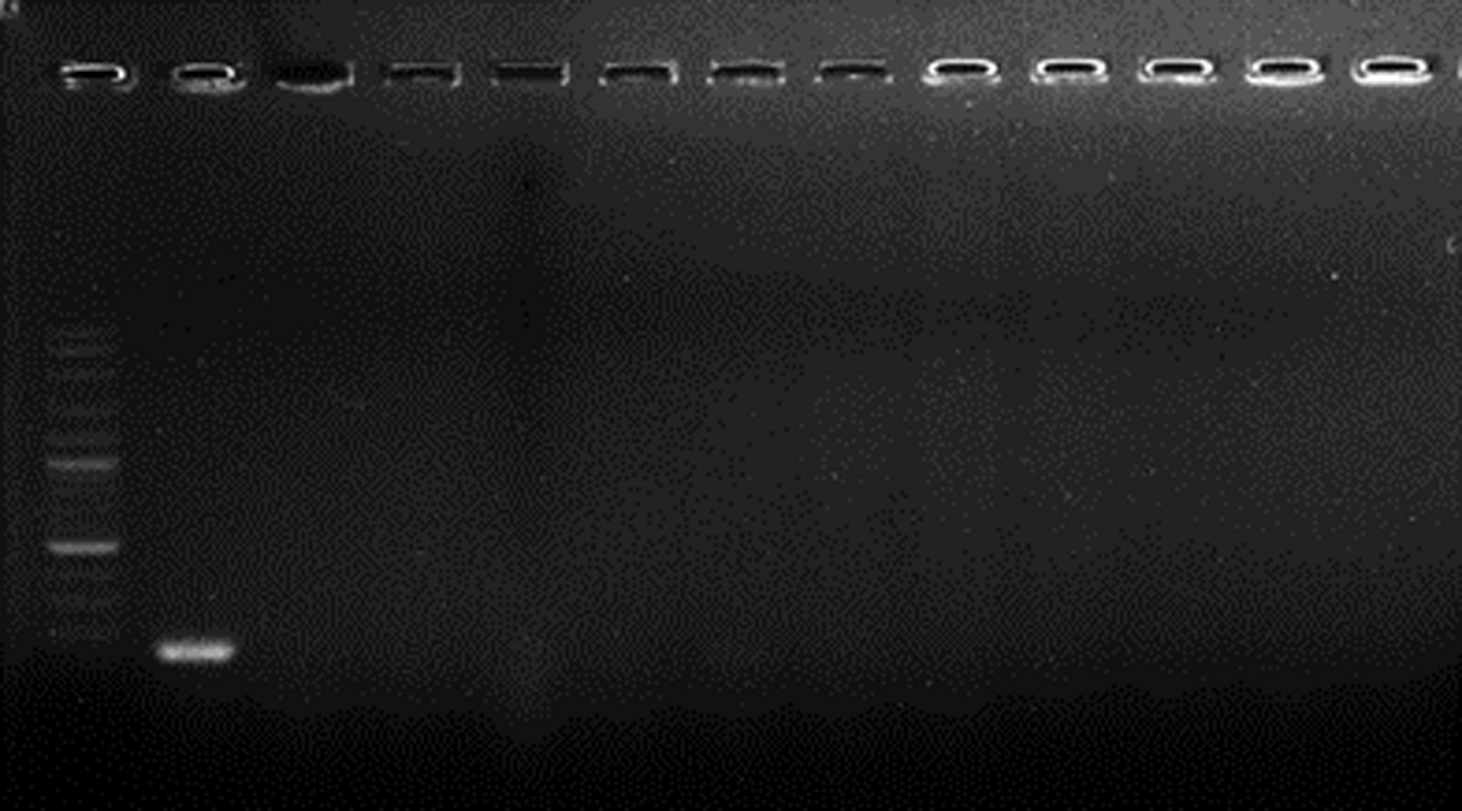
Detection of white spot syndrome virus in surviving and control shrimp samples by PCR. Lane 1, ladder; lane 2, positive control; lanes 3–6, survived shrimp samples; lanes 7–12, control shrimp samples; lane 13, negative control.

Based on the PCR and qPCR detection method described earlier using OIE primer pairs VP28 and VP28-140, all the samples that were challenged with white spot syndrome virus and survived were shown to be negative for the infection (Fig. 1).

### Transcriptome result

#### Sequencing and *de novo* assembly

cDNA libraries from mRNAs extracted from the hepatopancreas, haemolymph and muscle tissue of surviving WSSV-challenged shrimp and control shrimp were subjected to a run on the Illumina HiSeq 2000 sequencing instrument, resulting in 55,692,118 and 56,206,168 raw reads respectively. A total of 49,488,606 high-quality cleaned reads were obtained in the WSSV-challenged library with a total of 49,589,106 high-quality cleaned reads were obtained in the control library after removal of repetitive and low-quality reads. After *de novo* assembly by Trinity, a total of 43,730 unigenes with an average length of 810 bp and N50 length of 1,667 bp were obtained from the WSSV-challenged library. In the control library, a total of 44,755 unigenes with an average length of 760 bp and N50 length of 1,504 bp were obtained (Table 2). A total of 37,223 unigenes with an average length of 1,051 bp and N50 length of 1,878 bp were harvested from the combined reads of the two libraries.

**Table 2:** Summary reads of Illumina HiSeq 2000 in surviving WSSV-challenged and control giant tiger shrimp cDNA libraries.

	WSSV-challenged giant tiger shrimp	Control giant tiger shrimp
Total sequenced cDNA	55,692,118	56,206,168
Cleaned reads	49,488,606	49,589,106
Total unigene after assembly	43,730	44,755
Unigene average length	810	760
N50 length	1,667	1,504

**Figure 2:**
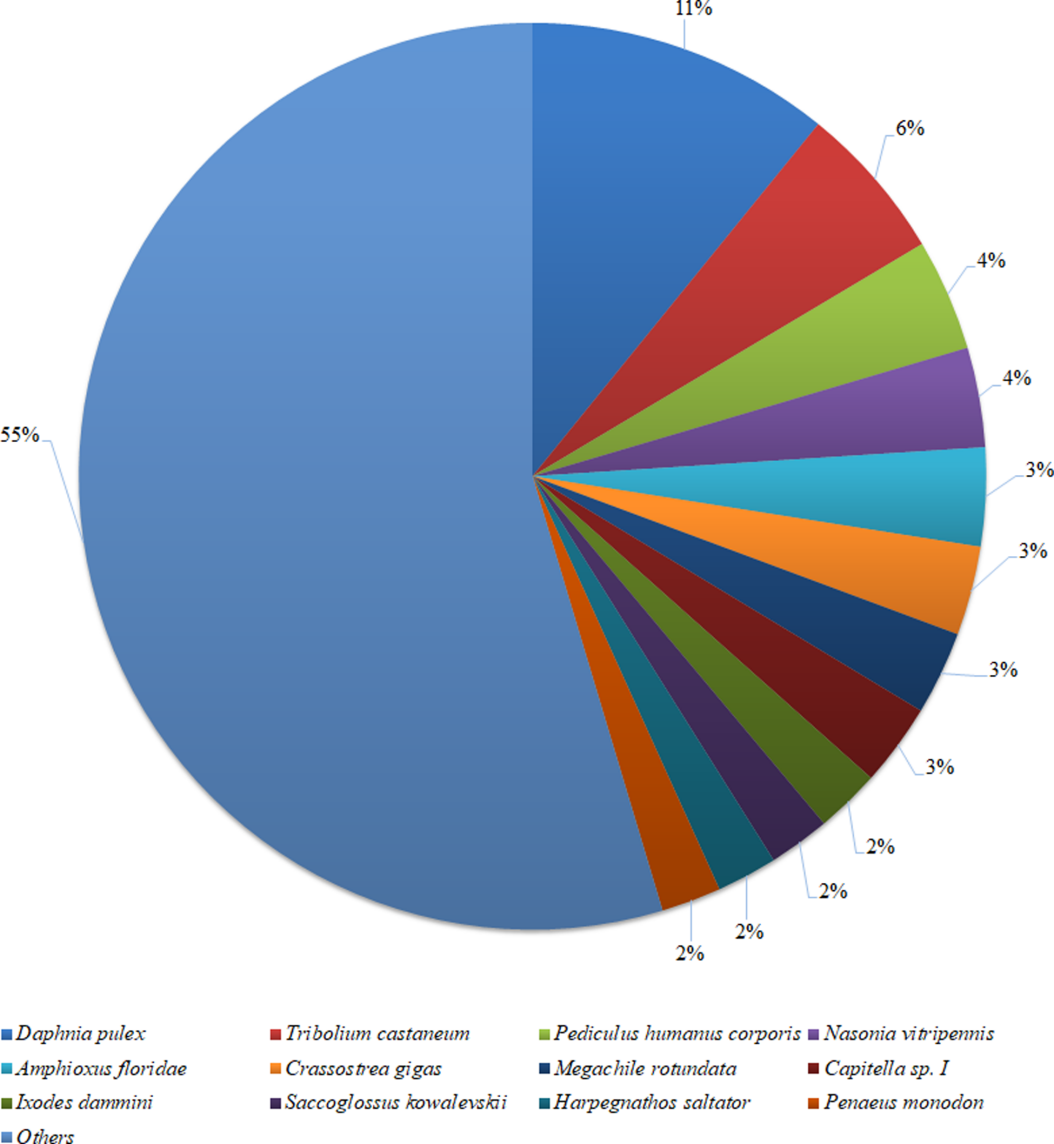
Species distribution of the BLASTX results. The figure shows the species distribution of the unigene BLASTX results against the NCBI non-redundant protein databases, with a cutoff *E* value of 10^−5^. Different colours represent different species. Only species with proportions of more than 1% are shown.

Based on a BLASTX similarity search of all unigenes against the NCBI non-redundant (NR) protein databases (cut off *e*-value <0.00001), 15,486 unigenes showed significant matches in the UniProtKB/Swiss-Prot database and 17,458 unigenes in the NR database. The species distribution of the best match result for each sequence is shown in Fig. 2. The *P. monodon* unigenes showed 10.8% matches with *Daphnia pulex* sequences, followed by *Tribolium castenum* (5.6%) and *Pediculus humanus corporis* (4.0%). All the read sequences obtained from the RNA-seq were submitted to NCBI Sequence Read Archive under BioProject ID: PRJNA480909; SRA accession: SRP153251; BioSample accessions: SAMN09652184, SAMN09652185.

### Transcriptome comparison between WSSV-infected and uninfected shrimp

#### Gene ontology assignments of differentially expressed genes

**Figure 3:**
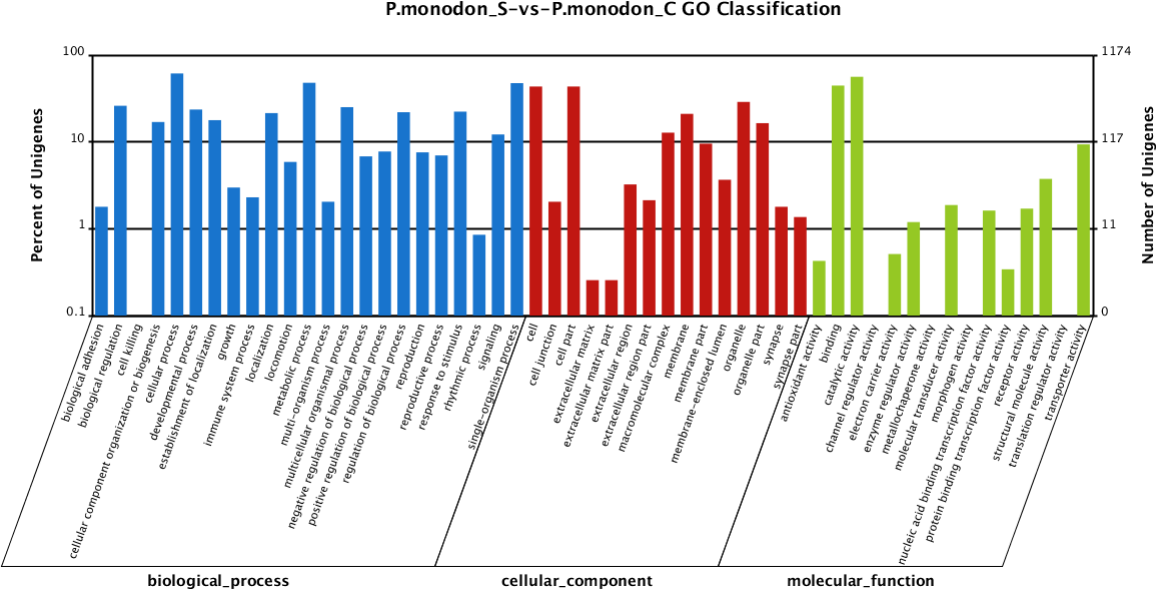
Gene Ontology (GO) classification of putative functions of unigenes from surviving WSSV-challenged and uninfected (control) giant tiger shrimp. The *x* axis shows subgroups of molecular functions from GO classification and the *y* axis shows the number of the matched unigenes.

In total, 4,572 unigenes (55.51%) of the differentially expressed unigenes between the survived WSSV-infected and control shrimp were mapped to biological processes, 2,234 unigenes (27.12%) were mapped to cellular components, and 1,431 unigenes (17.37%) were mapped to molecular functions (Fig. 3). Regarding the gene ontology (GO) assignment to biological processes in the differentially expressed genes, most were involved in cellular processes (15.77%), metabolic processes (12.31%) and single-organism processes (12.20%). GO assignments for the cellular component genes were associated with cells (22.83%), parts of cells (22.83%) and cell organelles (15.17%). Additionally, most of the GO assignments of the molecular function genes were associated with catalytic activity (46.12%) or binding (36.55%), with a smaller proportion associated with transporter activity (7.69%).

#### Identification of differentially expressed genes

**Figure 4:**
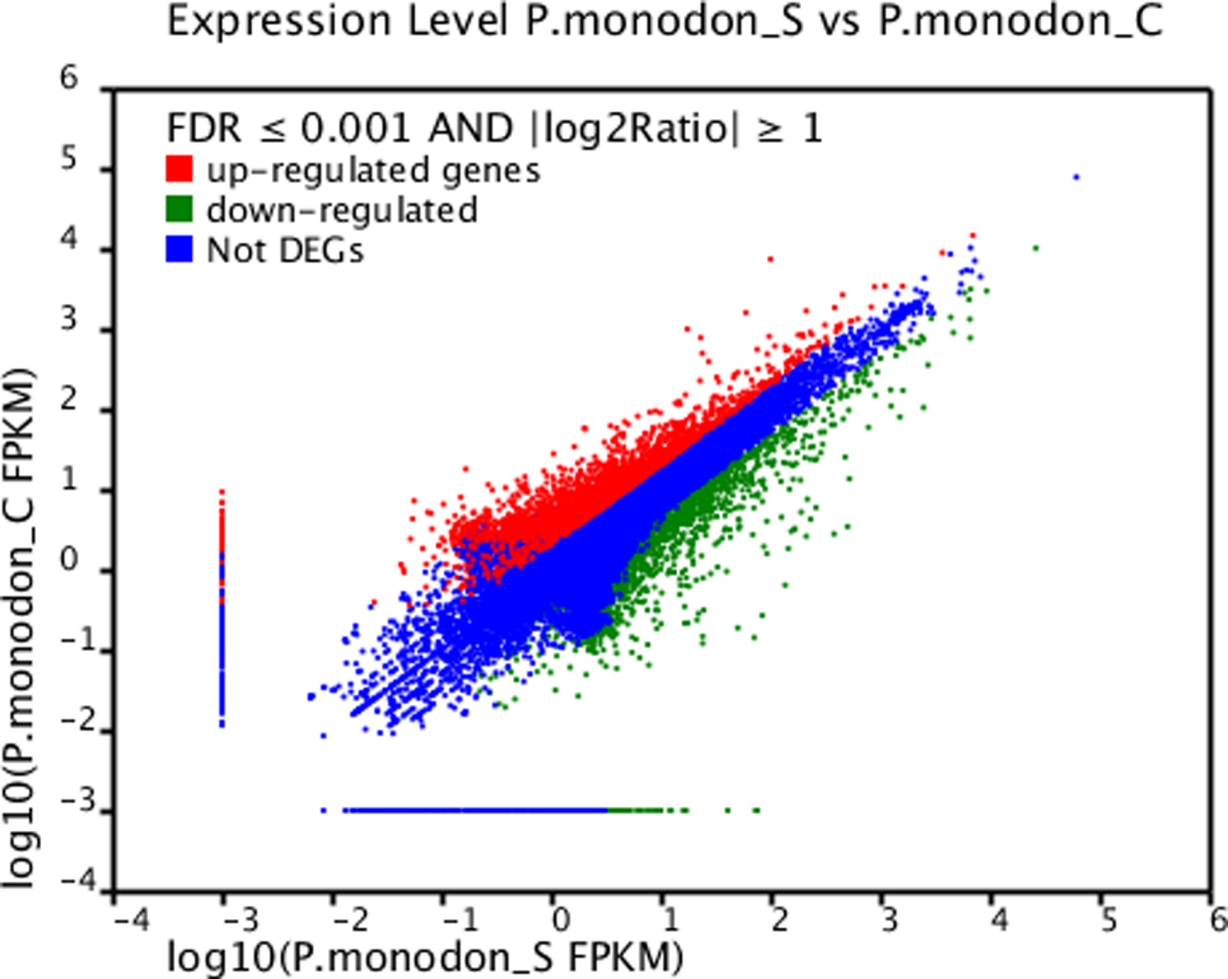
Scatter plot showing gene expression levels from giant tiger shrimp. Average FPKM values after WSSV-challenge correlated to average FPKM values for each gene in normal conditions.

**Figure 5:**
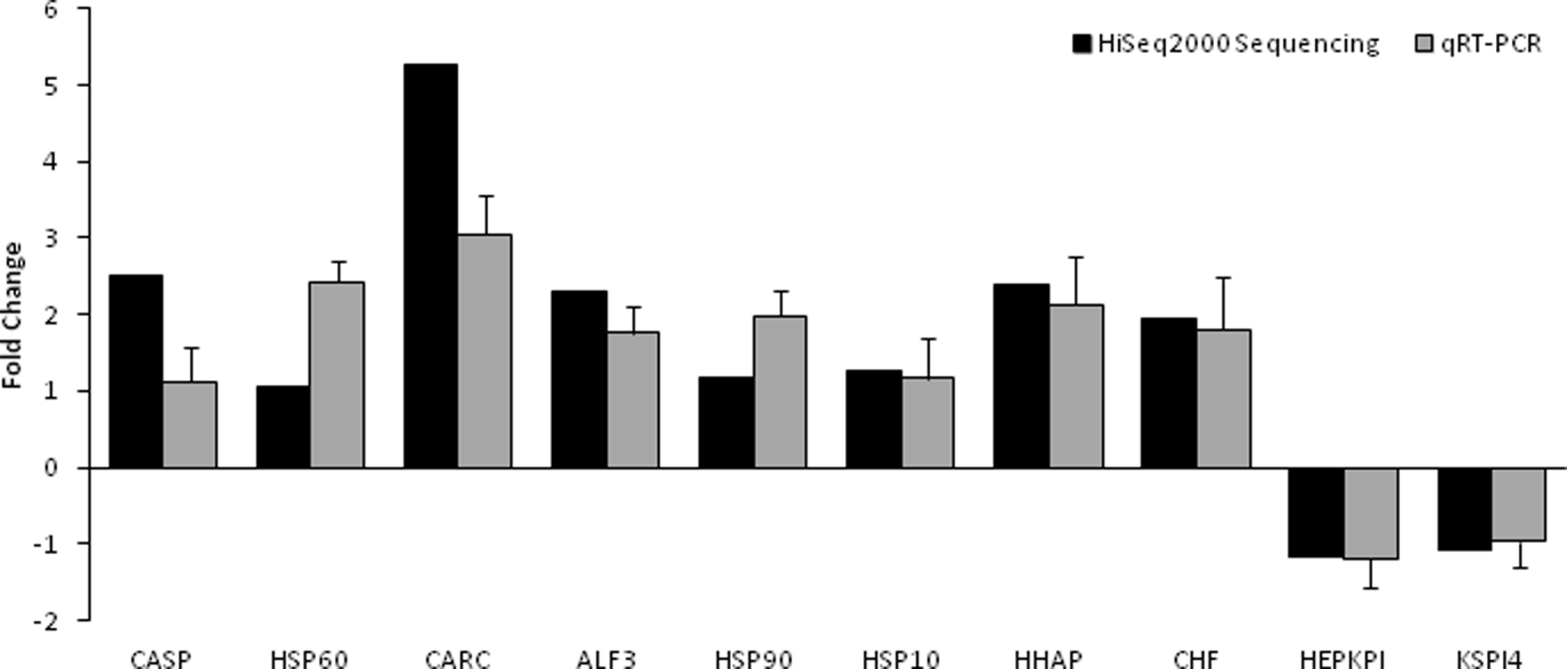
Comparison of expression profiles of selected genes as determined by Illumina HiSeq 2000 sequencing (black) and qRT-PCR (grey) in WSSV-challenged shrimp. Target gene abbreviations are as follows: CASP—caspase, HSP60—heat shock protein 60, CARC—carcinin, ALF3—anti-lipopolisaccharide factor-3, HSP90—heat shock protein 90, HSP 10—heat shock protein 10, HHAP—haemocyte homeostasis-associated protein, CHF—crustacean hematopoietic factor, HEPKPI—hepatopancreas kazal-type proteinase inhibitor 1A1 and KSPI4—kazal-type serine proteinase inhibitor 4. The results showed validation of the differential expression for each selected genes as determined by Illumina HiSeq 2000 sequencing and qRT-PCR between the survived WSSV-challenged shrimp and control group.

Based on significant differences in the expression of relative transcript abundance between the WSSV-challenged and uninfected control shrimp unigenes, 2,644 host genes were significantly up-regulated, and 2,194 genes were significantly down-regulated by infection with WSSV. A scatter plot was generated for FPKM values from the treatment group (Fig. 4). The qRT-PCR results confirmed the data obtained from the Illumina HiSeq 2000 sequencing analysis, showing similar trends in the up- and down-regulation of host genes (Fig. 5).

#### KEGG pathway analysis of the differentially expressed genes

All the differentially expressed genes related to virus infection of the host gene were characterized by mapping them against the referential canonical pathways in the Kyoto Encyclopedia of Genes and Genomes (KEGG) database. The most abundant categories were associated with amino sugar and nucleotide sugar metabolism, ubiquinone and other terpenoid-quinone biosynthesis, fatty acid metabolism, biosynthesis of secondary metabolites and folate biosynthesis (Table 3).

**Table 3:** KEGG pathway enrichment analysis for differentially expressed genes (DEGs) in WSSV- challenged giant tiger shrimp (*P* value < 0.05).

KEGG pathway	DEGs with pathway annotation
Amino sugar and nucleotide sugar metabolism	30 (1.4%)
Ubiquinone and other terpenoid-quinone biosynthesis	10 (0.47%)
Fatty acid metabolism	14 (0.66%)
Biosynthesis of secondary metabolites	86 (4.03%)
Folate biosynthesis	8 (0.37%)
Phagosome	27 (1.26%)
Cysteine and methionine metabolism	18 (0.84%)
Inositol phosphate metabolism	14 (0.66%)
Caffeine metabolism	8 (0.37%)
Glycerophospholipid metabolism	19 (0.89%)

### Candidate genes involved in *P. monodon* immune response

Based on the genes that were found to be differentially expressed in the WSSV-challenged shrimp compared to the uninfected controls, several genes were selected as candidate genes for biomarkers related to resistance in the survived WSSV-challenged *P. monodon*. Those genes include the high mobility group box b protein (HMGBb), tumor necrosis factor superfamily (TNFSF), c-Jun protein, as well as a series of Kazal type serine proteinase inhibitors including the haemocyte kazal type proteinase inhibitor and hepatopancreas kazal type proteinase inhibitor. In addition, we also discovered immune-related genes involved in shrimp defence against invading pathogens in various pathway such as MAPK signaling pathway, apoptosis pathway, toll-like receptors pathway and the prophenoloxidase activation system pathway. Genes associated with the host intracellular environment in response to WSSV were also identified (Table 4).

**Table 4:** Candidate genes selected in WSSV-challenged giant tiger shrimp.

Category	Homologous function	Fold changes in gene expression
MAPK Signalling Pathway	Heat shock protein 21	3.47
	Anti-lipopolysaccharide factor isoform 3	2.30
	chaperonin 10	1.25
Apoptosis Pathway	Caspase	2.49
	Cathepsin L	−1.21
Toll-like Receptor Pathway	Toll protein	1.02
	Crustin type 1	2.13
Prophenoloxidase Activation Pathway	C-type lectin	1.04
	Haemocyanin	6.21
	Haemocyte homeostasis-associated protein	2.38
Signal Transduction Pathway	Tumor necrosis factor superfamily (TNFSF)	1.34
	High mobility group box b protein (HMGBb)	1.75
	c-Jun Protein	1.54
Proteinase & Proteinase Inhibitors	Kazal type serine proteinase inhibitors (SPIs)	−1.08
	Haemocyte kazal type proteinase inhibitor	−1.64
	Hepatopancreas kazal type proteinase inhibitor	−1.18
Intracellular Genes	Plasmolipin	2.25
	FAD oxidoreductase	8.65
	G protein alpha subunit	2.47
	Peritrophin	−7.64
	Sodium/potassium-transporting ATPase subunit beta	−3.25

**Figure 6:**
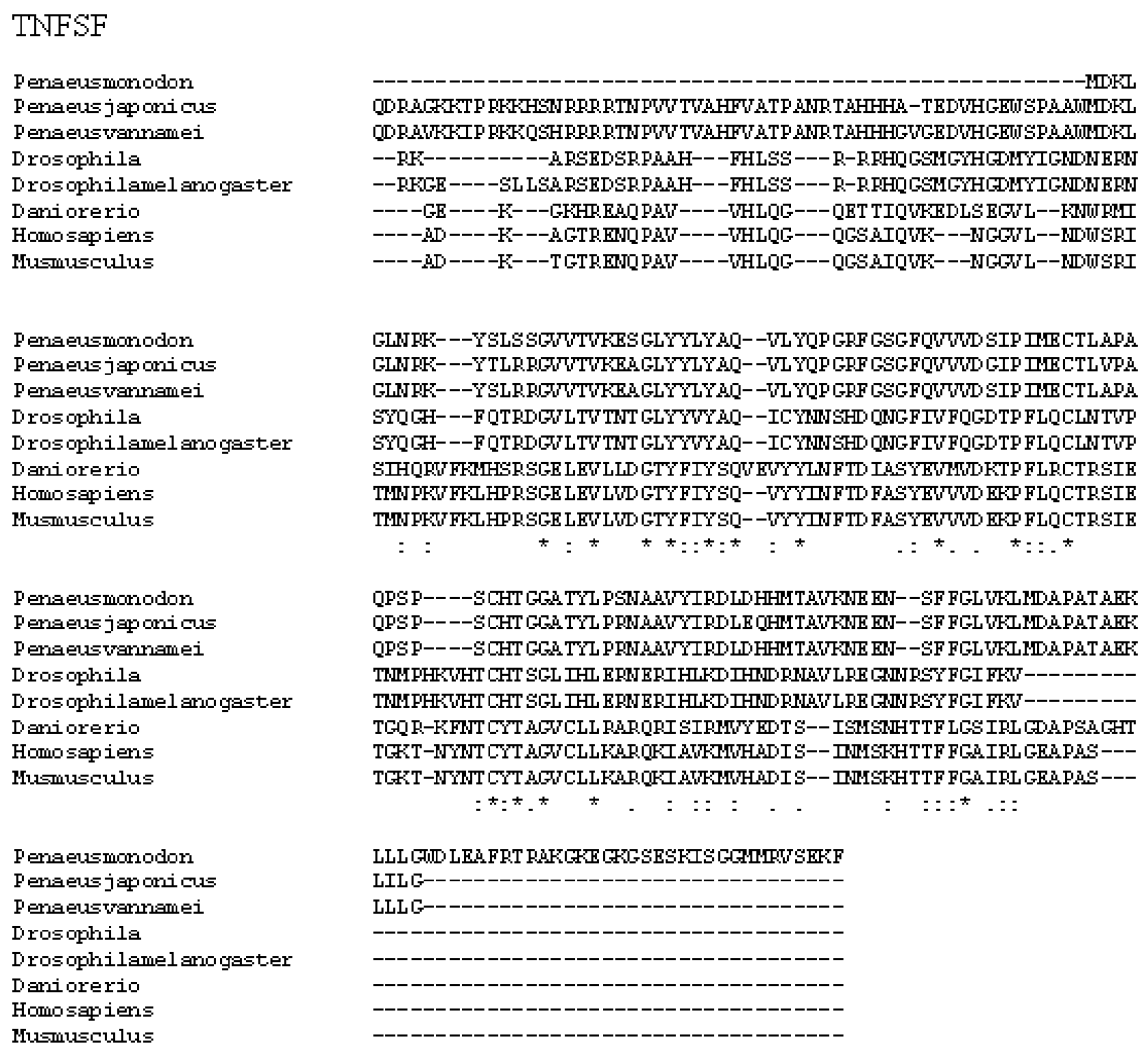
Multiple alignment analysis of amino acid sequences of PmTNF and its homologs. The fully conserved amino acid residues in these sequences are indicated by ‘*’. Conservation between groups of strongly similar properties are indicated in ‘:’. A ‘.’ indicates conservation between groups of weakly similar properties. The species names and GenBank accession numbers of TNF sequences used in this study are listed in Table 5.

**Figure 7:**
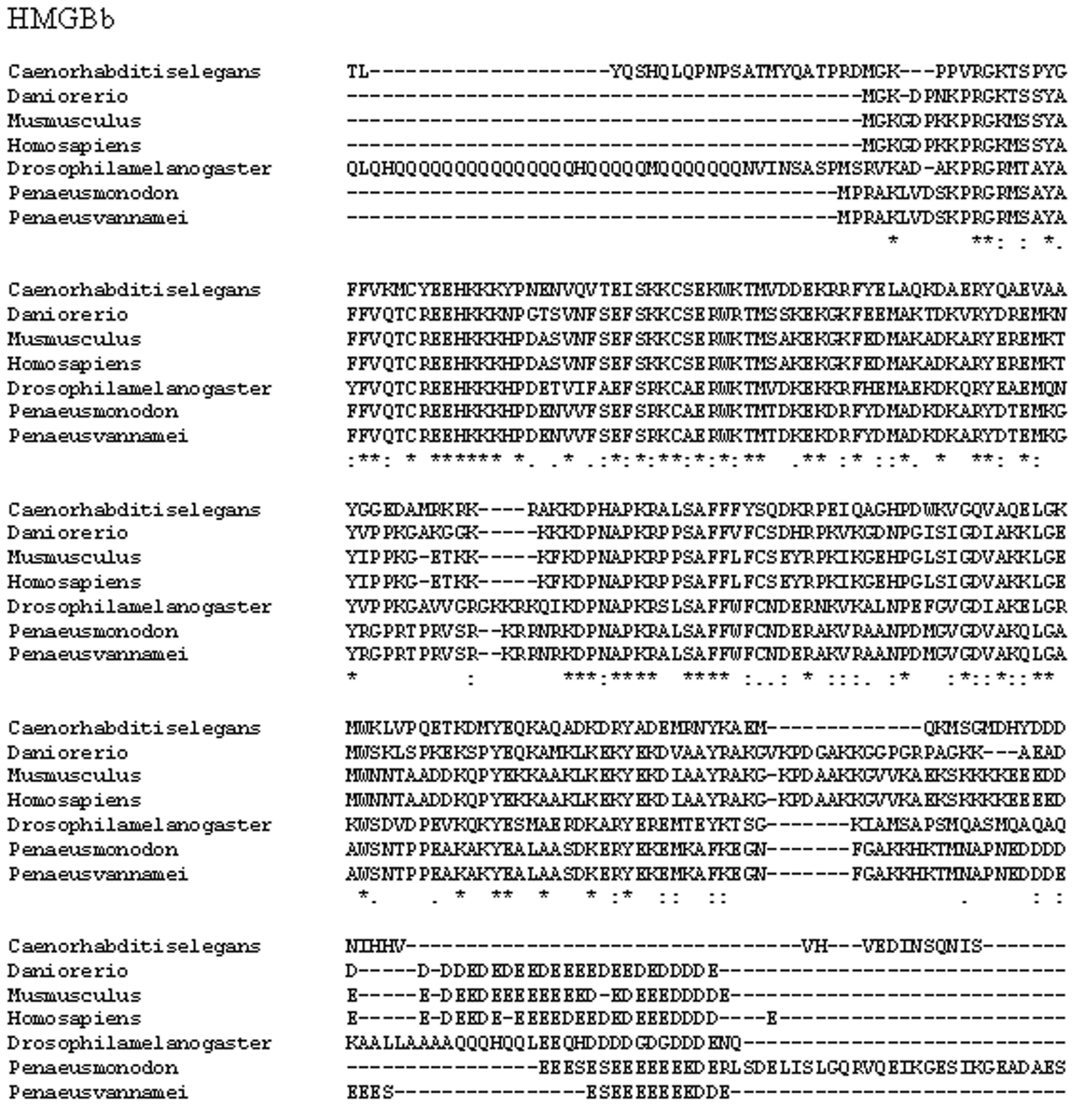
Multiple alignment analysis of amino acid sequences of PmHMGBb and its homologs. The fully conserved amino acid residues in these sequences are indicated by ‘*’. Conservation between groups of strongly similar properties are indicated in ‘:’. A ‘.’ indicates conservation between groups of weakly similar properties. The species names and GenBank accession numbers of HMGBb sequences used in this study are listed in Table 5.

**Figure 8:**
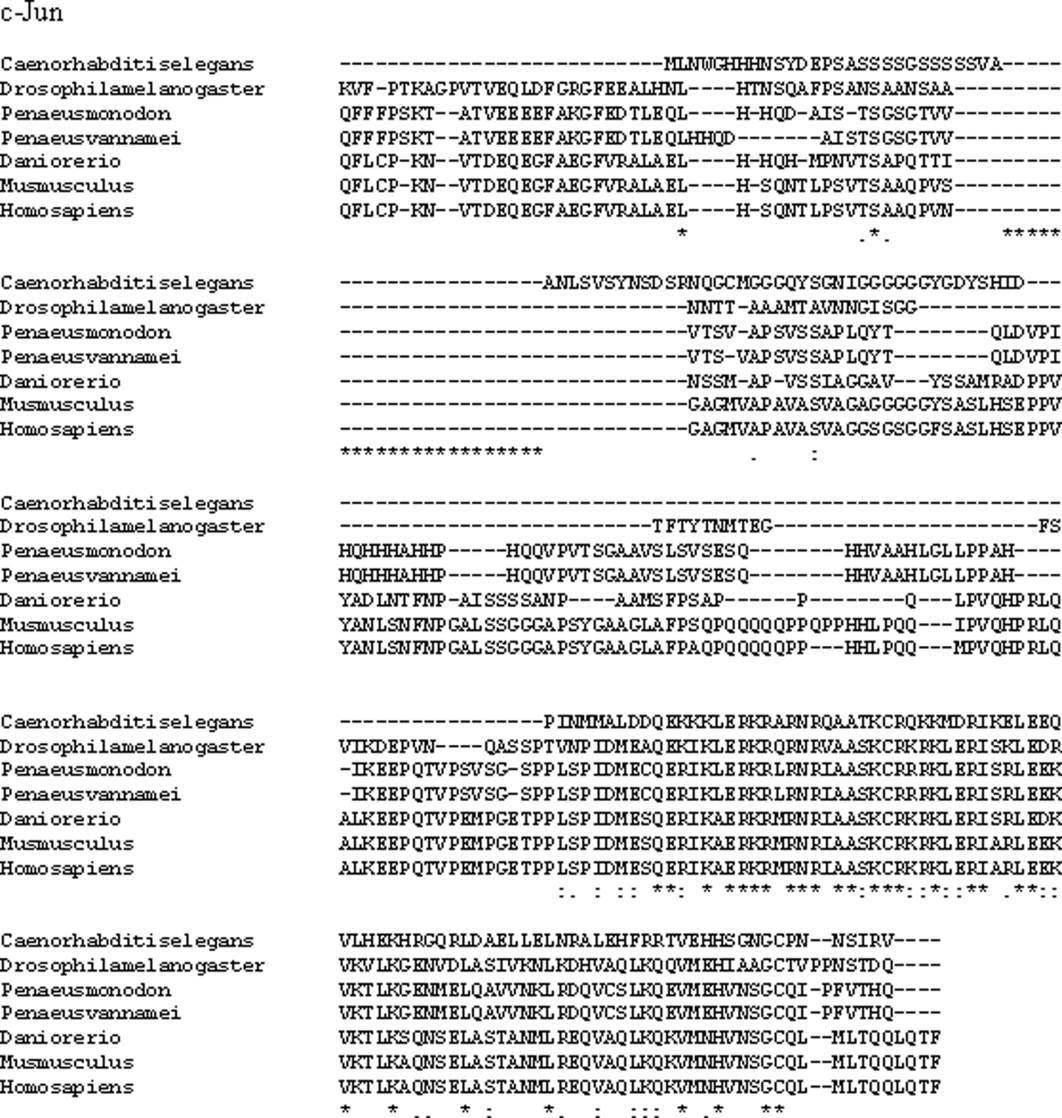
Multiple alignment analysis of amino acid sequences of PmcJun and its homologs. The fully conserved amino acid residues in these sequences are indicated by ‘*’. Conservation between groups of strongly similar properties are indicated in ‘:’. A ‘.’ indicates conservation between groups of weakly similar properties. The species names and GenBank accession numbers of c-Jun sequences used in this study are listed in Table 5.

**Figure 9:**
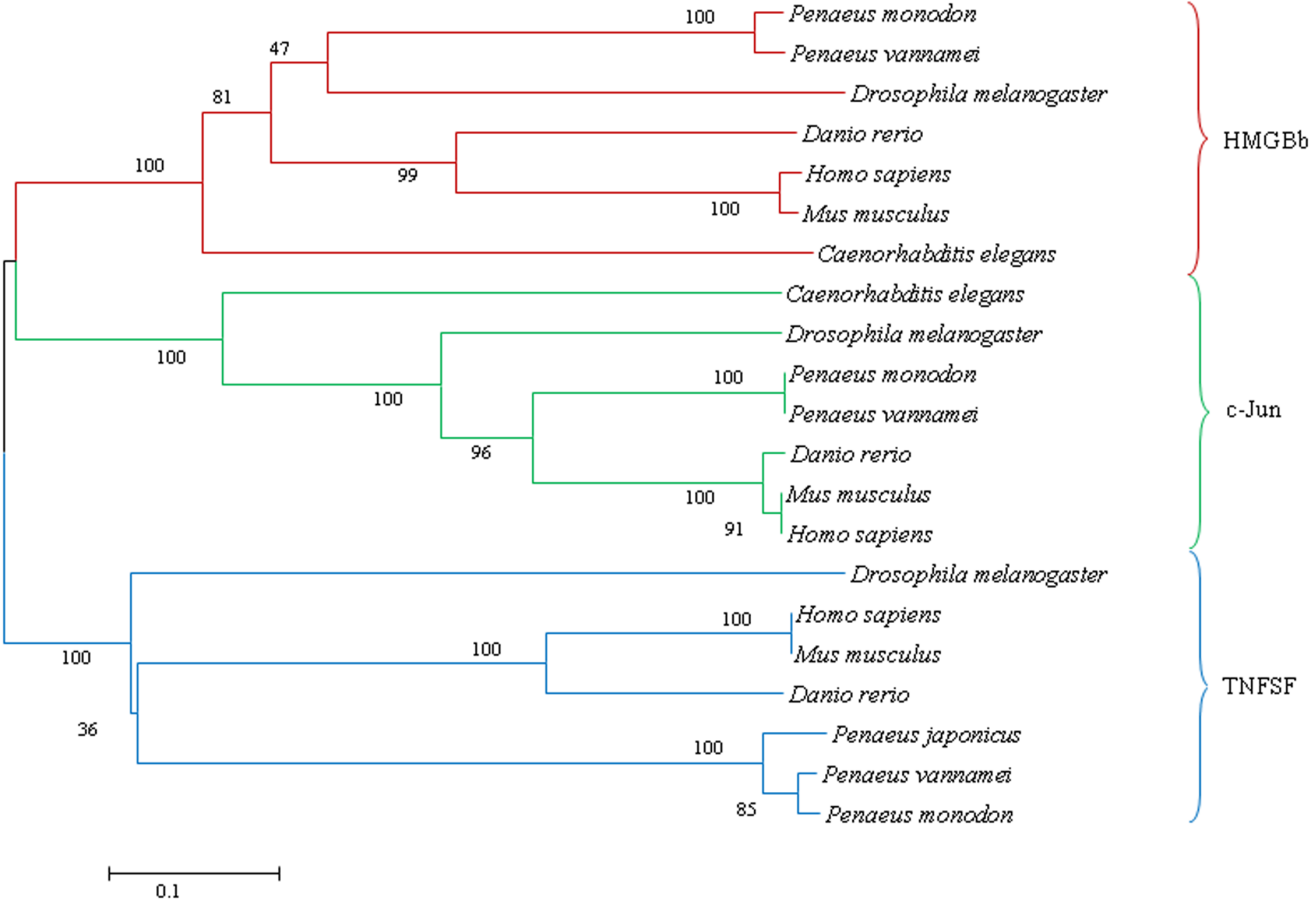
A phylogenetic tree of HMGBb, c-Jun and TNF homologs from *P. monodon* and other species were constructed using MEGA 6.0 with the neighbor-joining method. Numbers at treenodes refer to percent bootstrap values after 1,000 replicates. The bar (0.1) indicates the genetic distance. The accession numbers of the selected HMGBb, c-Jun and TNF sequences are listed in Table 5.

**Table 5:** Amino acid identity comparison of the PmTNF, PmHMGBb and PmcJun with other known homologues.

Gene	Species	GenBank accession number	Amino acid identity (%)
TNFSF	*Penaeus monodon*		–
	*Penaeus vannamei*	AEK86525.1	96.0
	*Penaeus japonicus*	BAJ10320.1	91.0
	*Drosophila melanogaster*	NP_724878.2	36.0
	*Danio rerio*	NP001108537.1	26.0
	*Mus musculus*	NP001171408.1	27.0
	*Homo sapiens*	NP001005609.1	27.0
HMGBb	*Penaeus monodon*		–
	*Penaeus vannamei*	ADQ43367.1	99.0
	*Drosophila melanogaster*	NP_727960.1	67.0
	*Danio rerio*	NP_001032501.1	56.0
	*Mus musculus*	NP_034569.1	58.0
	*Homo sapiens*	NP_002119.1	58.0
	*Caenorhabditis elegans*	NP_001022600.1	45.0
c-Jun	*Penaeus monodon*		–
	*Penaeus vannamei*	AIB53746.1	99.0
	*Drosophila melanogaster*	NP_476586.1	36.0
	*Danio rerio*	NP_956281.1	45.0
	*Mus musculus*	NP_034721.1	43.0
	*Homo sapiens*	NP_002219.1	43.0
	*Caenorhabditis elegans*	NP_001122643.1	40.0

### Characterisation, sequence analysis and immune response of *P. monodon* tumor necrosis factor, high mobility group box b protein and c-Jun protein

*P. monodon* TNF (PmTNF) gene was 1,177 bp long, containing a 453 bp ORF encoding 150 amino acids. Bioinformatics analysis of PmTNF sequence with the BlastP program revealed that the deduced amino acid sequence of PmTNF exhibited similarities with the tumor necrosis factor of other species (Fig. 6). PmHMGBb gene was 2,657 bp long, containing a 2,601 bp ORF encoding 866 amino acids. The deduced amino acid sequence of PmHMGBb exhibited similarities with the high mobility group box b of other species (Fig. 7). PmcJun gene was 1807 bp long, containing a 879 bp ORF encoding 292 amino acids. The deduced amino acid sequence of PmcJun exhibited similarities with the c-Jun of other species (Fig. 8).

Conserved motifs were observed between the gene sequences orthologs of PmHMGBb and PmcJun. Interestingly, the multiple sequence alignment of PmTNF between species revealed nonsynonymous mutations among its orthologs. To further investigate the nonsynonymous mutations observed in PmTNF orthologs have been shaped by positive selection pressure, the presence of sites under positive selection was calculated by the ratio of nonsynonymous to synonymous substitutions (dN/dS) per codon. However, the nonsynonymous polymorphic sites showed no evidence of positive selection.

A phylogenetic tree was also constructed to gain insights into the evolutionary relationship between the various animal tumor necrosis factor, high mobility group box b and c-Jun sequences (Fig. 9). In general, the tree shows that all the shrimp TNFSF, HMGBb and c-Jun each were clustered within a single clade. Amino acid identity comparison of PmTNF, PmHMGBb and PmcJun with other known homologues is shown in Table 5.

**Figure 10:**
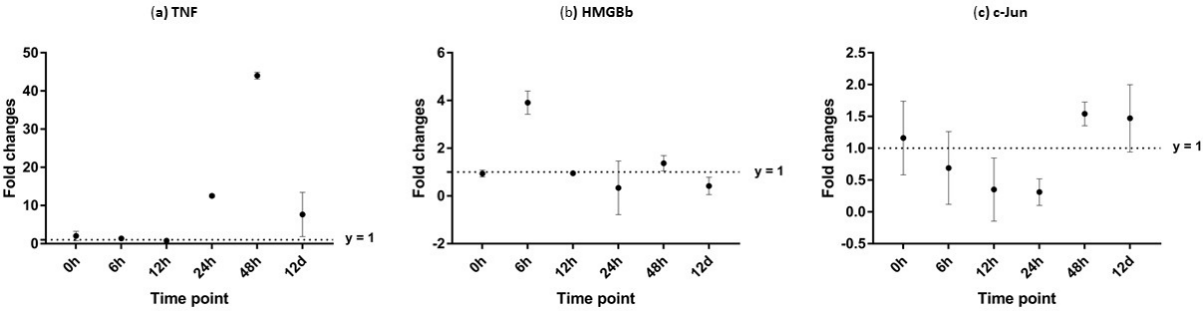
Analysis of gene expression profile in hepatopancreas of WSSV-challenged giant tiger shrimp by microfluidic dynamic array at 0, 6, 12, 24, 48 h and 12 days post-injection in (A) TNF, (B) HMGBb and (C) c-Jun. Each dot represents the mean fold change of the normalized expression levels of the replicates (*N* = 3). Data (mean ± SE) with (*) are significant at (*p* < 0.05). Axis *y* = 1 indicates control group at log 0.

**Figure 11:**
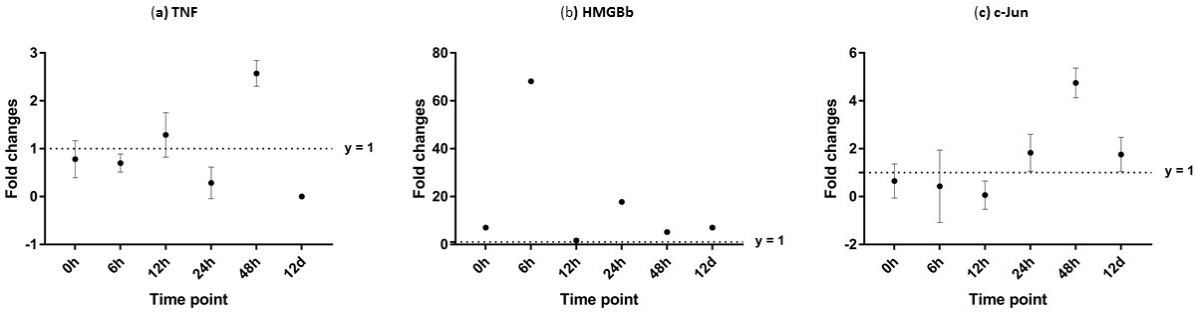
Analysis of gene expression profile in haemocytes of WSSV-challenged giant tiger shrimp by microfluidic dynamic array at 0, 6, 12, 24, 48 h and 12 days post-injection in (A) TNF, (B) HMGBb and (C) c-Jun. Each dot represents the mean fold change of the normalized expression levels of the replicates (*N* = 3). Data (mean ± SE) with (*) are significant at (*p* < 0.05). Axis *y* = 1 indicates control group at log 0.

After challenged with WSSV, PmTNF and PmHMGBb in giant tiger shrimp hepatopancreas showed some changes in expression level (Fig. 10). In HMGB, the expression level peaks at 6 hpi and decreased afterwards until it reached same expression as of the control group. The gene expression level in TNFSF increased from 0 hpi until it reached highest expression level at 48 hpi with WSSV treatment. In haemocytes of challenged shrimp, PmHMGBb gene expression started to increase immediately after WSSV infection and reached its peaks at 6 hpi by 68 fold, before its reached the lowest expression at 12 hpi. This gene expression started to increase again at 24 hpi by 17 folds and decrease again by 5 folds at 48 hpi. The gene expression level of PmTNF slightly decreased below the control group shrimp at 0 hpi and 6 hpi before fairly increased above the control shrimp group at 12 hpi. The expression of TNF gene in infected shrimp then decreased again below the control shrimp expression level at 24 hpi and reach it maximum expression level at 48 hpi by 2.5 fold. In c-Jun, the gene was down-regulated below control shrimp group gradually from 0 hpi to 12 hpi but started to increase at 24 hpi until it reached its peak at 48 hpi by 4.7 fold (Fig. 11).

**Figure 12:**
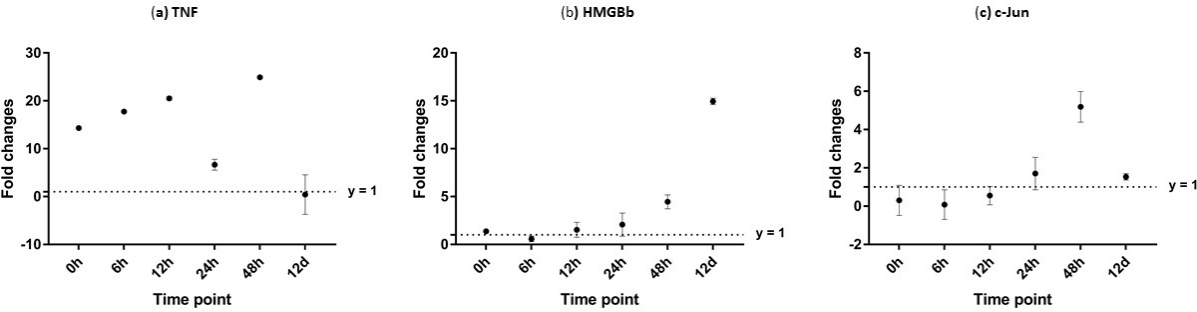
Analysis of gene expression profile in muscle tissue of WSSV-challenged giant tiger shrimp by microfluidic dynamic array at 0, 6, 12, 24, 48 h and 12 days post-injection in (A) TNF, (B) HMGBb and (C) c-Jun. Each dot represents the mean fold change of the normalized expression levels of the replicates (*N* = 3). Data (mean ± SE) with (*) are significant at (*p* < 0.05). Axis *y* = 1 indicates control group at log 0.

Additionally, immune response of both PmTNF and PmcJun were also observed in muscle tissue of infected shrimp. TNF gene expression level started to increase from 0 hpi until it reached maximum expression 48 hpi by 24 fold. However, a sudden decreased in expression level of TNF can be observed after WSSV infection at 24 hpi. C-Jun protein was down-regulated below control shrimp group from 0 hpi to 12 hpi but started to increase at 24 hpi until it reached its peak at 48 hpi by 5 fold (Fig. 12).

## Discussion

The WSSV is a major disease in penaeid shrimp aquaculture, which has caused massive cumulative losses to the industry (estimated at 6 billion USD) since its emergence in 1992 (Lightner et al., 2012). Until now, there has been no effective treatment available, and the industry has mainly relied on good management practice to contain and reduce infection (Kongkeo, 2005; Verbruggen et al., 2016).

Various studies have been carried out on virus infection pathways and the shrimp immune system (Robalino et al., 2007; Zeng & Lu, 2009; Chen et al., 2013; Xue et al., 2013). However, most of the researches have been conducted on shrimp three days after they have been infected with WSSV. The present study, in contrast, investigated shrimp that had survived for at least 12 days after challenged by WSSV and the interactions between WSSV and the host intracellular environment. This gave an opportunity to compare, via transcriptome analysis, the gene expression in shrimp that had survived WSSV infection with that of healthy shrimp, to gain a better understanding of the host-virus interactions. In the process, five potential candidate genes for disease resistance against WSSV were identified: HMGB, TNFSF, c-Jun and a series of Kazal type serine proteinase inhibitors (haemocyte kazal type proteinase inhibitor and hepatopancreas kazal type proteinase inhibitor). The present study is, to our knowledge, the first to discover HMGB and c-Jun in *P. monodon*.

HMGB plays an important role in the signal-transducing antiviral immune response (Yanai, Ban & Taniguchi, 2012). Indeed, it has been recognized as the universal sentinel of nucleic-acid-mediated innate immune responses (Yanai et al., 2009). This protein can be secreted into the extracellular environment as a signaling molecule when cells are under stress. It also acts as DNA chaperones influencing multiple processes in chromatin such as transcription, replication, recombination, DNA repair and genomic stability (Stros, 2010). In mammals, there are four members of the HMGB family, all of which (HMGB1-4) function as chaperones influencing multiple processes in chromatin including transcription, replication, recombination, DNA repair and genomic stability (Stros, 2010). There are various members of HMGB in lower vertebrates and invertebrates (Rao & Su, 2015). In fishes, HMGB1, HMGB2 and HMGB3 are present in cartilaginous fish and bony fish (Moleri et al., 2011), while two types of HMGB are found in white shrimp (*P. vannamei*): HMGBa and HMGBb (Chen et al., 2011).

The multiple alignment of HMGBa and HMGBb in white shrimp is similar to that of HMGB1 and HMGB2 in other species, with the presence of two DNA binding domains (A box and B box) as well as a tail (Chen et al., 2014a; Chen et al., 2014b). A study by Chen et al. (2014a) and Chen et al. (2014b) into white shrimp found that HMGBb expression levels were up-regulated when induced by a pathogen-associated molecular pattern (PAMP). They suggested that the release of HMGBa and HMGBb occurs naturally during cell necrosis, and that it occurs in shrimp haemocytes in response to PAMP. The expression of HMGBb in this present study was elevated at 6 h post infection with WSSV. The result indicates that this gene plays an important role as the first line of defense in shrimp innate immunity as pattern recognition receptors (PRRs). Further research is required on the role of this gene and into the mechanisms through which it acts in innate immunity in shrimps, particularly in *P. monodon* against WSSV infection.

The Janus family tyrosine kinase and signal transducer and activator of transcription (JAK/STAT) signaling pathway has been proven to be very important in antiviral immunity in both vertebrates and invertebrates (Glenney & Wiens, 2007; Sonar & Lal, 2015). However, little is known about the function of this signaling pathway in the antiviral immunity of shrimp; and in particular about the TNFSF, one of the potential STAT regulator genes (Wen et al., 2014). This gene activates caspase via the extrinsic pathway by promoting the oligomerization of caspases at the intracellular domain of the membrane-spanning TNFR (Salvesen, 2008; Menze et al., 2010). A high level of expression of MjTNF gene was observed following stimulation with peptidoglycan and polycytidylic acid in lymphoid organs cells. A high expression level of MjTNF was also observed *in vivo* 2 h and 4 h after stimulation with lipopolysaccharide and *Vibrio penaeicida* respectively (Mekata et al., 2010). LvTNFSF also responded to viral infection in a similar manner to MjTNFSF. LvTNFSF was upregulated at 3 and 24 h post-injection with WSSV in the hepatopancreas, suggesting that LvTNFSF may participate in host immune responses against pathogens, especially Gram-positive bacteria and viruses (Wang et al., 2012).

To date, only a small number of TNFSF members have been identified in shrimp, including in *P. japonicus* (Mekata et al., 2010) and *P. vannamei* (Wang et al., 2012). A homology analysis of the kuruma shrimp TNF (MjTNF) showed 30.7% and 26.7% identities with fruit fly (*Drosophila melanogaster*) Eiger and human (*Homo sapiens*) ectodysplasin A (Mekata et al., 2010). Ectodysplasin-A plays an important role in mammalian development (Harris et al., 2008). Our discovery of giant tiger shrimp TNF will provide more information and should lead to a better understanding of shrimp inflammatory responses. The sequence polymorphisms detected in PmTNF discovered in the present study could have consequences on the differential susceptibility of the survived giant tiger shrimp. A high polymorphism in two AMP families (Cg-Defs and Cg-Prp), in Cg-Toll and in glutathione reductase genes was also discovered in *Crassostrea gigas,* the Pacific oyster, that showed resistance to summer mortalities (Schmitt et al., 2013). Similarly, resistance to Vibrio infection was observed in *Scylla paramamosain*, green mud crab, with different variants of the Sp-Toll gene (Lin et al., 2012). The presence of the polymorphic sites found in PmTNF might contributed to the improvement of the disease resistance to WSSV in giant tiger shrimp. However, more work with larger sample size should be oriented to correlate the contribution of this gene towards improved immune response in giant tiger shrimp.

C-Jun, a member of the Jun family along with JunB and JunD, is a major substrate of c-Jun N-terminal kinase (JNK). It participates in regulating gene transcription in response to various stimuli, including cytokines, stress signals, and bacterial and viral infection (Yao et al., 2015). C-Jun was first reported in 1987 (Maki et al., 1987) and has recently been reported to be involved in WSSV gene transcription (Shi et al., 2012; Yao et al., 2015). Yao et al. (2015) found that, during the process of WSSV infection, the transcription levels of *P. vannamei* c-Jun (Lvc-Jun) were up-regulated, suggesting that WSSV infection could enhance both the expression and phosphorylation levels of Lvc-Jun. The same authors further stated that the increased level of Lvc-Jun along with the aggravation of viral infection indicated a notable positive correlation between Lvc-Jun activation and viral infection. Another study by Li et al. (2015), on the interaction of *P. vannamei* encoding the full-length c-Fos protein (Lvc-Fos) and Lvc-Jun, found that silencing of Lvc-Fos or Lvc-Jun in shrimp caused lower mortality and virus loads under WSSV infection, suggesting that Lvc-Fos and Lvc-Jun could be engaged in WSSV replication and pathogenesis. In contrast, our findings suggested that, while c-Jun was upregulated in surviving WSSV-infected shrimp, the activation of this gene in cases of WSSV infection may trigger a sequence of immune-related genes and immune pathways to help the shrimp to survive the disease. In summary, more studies on the interaction of immune-related genes are needed to elucidate their role in immune responses against pathogens, particularly in surviving shrimp.

An optimal environment is needed for a virus to replicate in a host cell. Virus will dwell within a host cell and altered the host cell pathways by making it beneficial for virus replication (Sanchez & Lagunoff, 2015). In response to the presence of virus, the host cellular environment will deteriorate, e.g., through draws on energy for anabolic reactions, demand for essential nutrients, and accumulation of non-host proteins, making it less conducive for viral replication (Verbruggen et al., 2016). Changes of intracellular environment in host cells to reduce the ability of virus to replicate was essential for survival (Mothes et al., 2010). In the present study, the host intracellular environment response to WSSV infection was observed and the specific genes involved were also recognized. Those genes include plasmolipin, G protein alpha subunit and peritrophin.

Plasmolipin is a membrane-bound 18-kDa proteolipid protein and consists of four transmembrane segments (Miller et al., 2008). It is an amphipathic protein, participates in transmembrane ion movement including H^+^, Ca^2+^ channels, and possibly in Na^+^, K^+^-ATPase transport function (Cochary et al., 1990). The biological function of plasmolipin is not known; but the *in vitro* formation of a voltage dependent K^+^ channel by plasmolipin suggests its ion channel function *in vivo* (Fischer & Sapirstein, 1994). Plasmolipin proteins were first reported from the canine and bovine kidney plasma membranes (Pérez, Puertollano & Alonso, 1997) and its homologues are found in ion homeostasis-dependent tissue, such as the apical surface of the kidney tubular cells and the myelin sheaths of the nervous system in the brain (Fischer & Sapirstein, 1994). In crustaceans, plasmolipin was first reported in *P. monodon* and is abundance in most tissue (Vatanavicharn, Pongsomboon & Tassanakajon, 2012).

A study by Miller et al. (2008) on *Mus caroli*, the Asian wild mouse, confirmed that plasmolipin (PLLP; TM4SF11), is a receptor for *M. caroli* endogenous retrovirus (McERV) but was not expressed in the mouse cell types. In *P. monodon*, two isoforms of plasmolipin, *Pm* PLP1 and *Pmp* LP2, was upregulated in haemocytes after infection with yellow head virus (YHV) and WSSV (Vatanavicharn, Pongsomboon & Tassanakajon, 2012). They proposed that *Pm* PLP in haemocytes may be required for the viral entry into shrimp haemocytes. In addition, *PM* PLP1 was postulated to be an YHV receptor, but with no evidence after a gene knock-down by dsRNA. In contrast, in this present study, plasmolipin was upregulated in the survived-WSSV shrimp, suggesting its potential role in defense response. However, the function of this gene needs further evaluation to verify it involvement in shrimp immune response.

Heterotrimeric guanine nucleotide binding proteins (G proteins) plays a vital role in transmembrane signaling process as they mediate the effects of neurotransmitters, numerous hormones or sensory stimuli by coupling their transmembranous receptors to various effectors like enzymes and ion channels (Wettschureck & Offermanns, 2005). G protein, activated by G protein–coupled receptors (GPCRs), are composed of three subunits, namely α, β, and γ (Offermanns & Simon, 1996). Activation of G protein α subunit will release guanosine diphosphate (GDP) and bind guanosine triphosphate (GTP) to G_α_, resulting in dissociation of the G_αβγ_ heterotrimer. Both G_βγ_ and GTP–G_α_ can activate downstream effectors (Kehrl, 1998). G protein α subunit is a part of chemokine receptors signaling that are widely expressed on a variety of immune cells (Bennett, Fox & Signoret, 2011). The biological function of these receptors are based on the receptors itself, either constitutive or inflammatory, predominantly involved in development and homeostasis, or in host response to infection (Johnson et al., 2005). These proteins are widely investigated in vertebrates (Du & Macara, 2004; Wettschureck & Offermanns, 2005; Tsvetanova, Irannejad & Zastrow, 2015; Kamp, Liu & Kortholt, 2016; Toro-Tapia et al., 2017).

In invertebrates, researches on G proteins mainly focus on neurotransmission, energy metabolism, longevity and stress resistance, germ cell migration and developmental regulation (Dong & Zhang, 2012). However, Dong & Zhang (2012) also reported limited researches on immune function of these proteins in invertebrates. In *Caenorhabditis elegans*, the roundworm, G protein-coupled receptors (GPCRs) have been demonstrated to be involved in the regulation of the innate immune response via neural and non-neural mechanisms (Liu & Sun, 2017). The (GPCR) DCAR-1 in *C. elegans* was revealed being required for the response to fungal infection and wounding (Zugasti et al., 2014). DCAR-1 acted in the epidermis to regulate the expression of antimicrobial peptides via p38 mitogen-activated protein kinase pathway. A putative GPCR found in *Procambarus clarkii*, the red swamp crayfish, HP1R gene, was required to defend against bacterial challenge (Dong & Zhang, 2012). The silencing of HP1R gene by RNA interference in crayfish demonstrated high bacterial burden and decreased total haemocytes count in response to bacterial challenge. A study by Xu et al. (2017) on G protein alpha signaling in Arabidopsis demonstrated that Arabidopsis Gα (GPA1) is a key component of a new immune signaling pathway activated by bacteria-secreted proteases. Therefore, the upregulation of the G protein α subunit established in the present study is the indication of response of innate immunity in crustacean to viral infection. However, this remains speculative as more research is needed to confirm its functions in crustacean innate immune system.

According to a study by Xie et al. (2015), the *P. vannamei* peritrophin-like protein (*Lv*PT) was found to interact with VP37, an envelope protein of the white spot syndrome virus. Further studies using the yeast two-hybrid (Y2H) library that was constructed using cDNA obtained from the stomach and gut of *P. vannamei* revealed that *Lv*PT could also interact with other WSSV envelope proteins such as VP32, VP38A, VP39B, and VP41A. VP37, found on the outside of the virion WSSV membrane protein complex (Chang et al., 2010), played a major role in WSSV infection (Wu, Wang & Zhang, 2005). This envelope protein interacts with receptors or assisting proteins on the peritrophic membrane (PM), possibly peritrophin, that can enable the WSSV to break through the physical barrier of the PM (Xie et al., 2015). PM only allows particles smaller than 20 nm to pass through the membrane (Martin et al., 2006), wherein, the size and length of WSSV is 70–150 nm and 250–380 nm, respectively (Lu et al., 1997). The PM aids in digestion and forms a protective barrier to prevent the invasion of bacteria, viruses and parasites (Lehane, 1997).

Peritrophin is a type 3 protein of the PM matrix that has been extensively studied in various organisms such as *Tribolium castaneum*, the red flour beetle (Jasrapuria et al., 2010), *Lucilia cuprina*, the green bottle fly (Elvin et al., 1996), *Spodoptera litura*, the common cutworm (Chen et al., 2014a; Chen et al., 2014b), *Anopheles gambiae*, African malaria mosquito (Shen & Jacobs-Lorena, 1998), *Eriocheir sinensis*, Chinese mitten crab (Huang et al., 2015) and *Exopalaemon carinicauda*, ridgetail prawn (Wang et al., 2013). This secretory protein was mainly expressed in the stomach and gills of *Penaeus chinensis*, the Chinese white shrimp (Du et al., 2006). The discovery of a peritrophin-like gene (*Es*PT) obtained from *E. sinensis* revealed that it could bind to different microbes and enhanced the clearance of *Vibrio parahaemolyticus in vivo* (Huang et al., 2015). However, in *E. carinicauda*, a peritrophin-like protein (*Ec*PT) might be involved in WSSV infection as silencing of *Ec*PT by dsRNA interference led to a higher survival rate of shrimp against WSSV challenge (Wang et al., 2013). Hence, the downregulation of peritrophin gene observed in this present study, might indicate the immune response of shrimp against WSSV infection and possibly increase the survival rate.

In the present study, the shrimp surviving at 12 days post post-infection were found to be clear of the virus. Venegas et al. (2000) demonstrated a quasi-immune response in *P. japonicus* that survived for 32 days after a series of WSSV infections. In this case, the shrimp that were reared collectively and those reared individually were 72% and 100% WSSV negative respectively when tested by PCR. Venegas et al. (2000) also hypothesized the presence of a ‘neutralizing factor’ (or non-specific binding factor) in the haemolymph of shrimp that survived after 17 days of the challenge with WSSV. The neutralizing factor could not be detected at the end of the 17-day survival period, but it again emerged following secondary exposure to the virus. However, the authors could not confirm the phenomenon of acquired resistance or a ‘quasi-immune response’ in *P. japonicus* to WSSV.

Clearance of WSSV was also observed by PCR in an experimentally injected giant freshwater prawn (*Macrobrachium rosenbergii)* (Sarathi et al., 2008). The giant freshwater prawn is known to act as an asymptomatic carrier for the virus (Hossain et al., 2001). Sahul Hameed, Xavier Charles & Anilkumar (2000) found that WSSV pathogenicity for juveniles and adults of *M. idella*, *M. lamerrae*, and *M. rosenbergii* pointed to the former two species being more susceptible to WSSV infections than *M. rosenbergii*, which has a high level of tolerance for the disease. *Macrobrachium rosenbergii* recovered and survived without any mortality at five days post-infection over the 100-days of the experiment. However, the mechanism of resistance involved in this species remains unknown. Thus, further work is needed to determine the exact mechanisms of clearance and resistance against WSSV.

## Conclusion

The utilization of transcriptomic studies in the present study could help the development of genomics toolkits and assist stock improvement in the giant tiger shrimp by marker assisted selection (MAS). The discovery of HMGB, TNFSF and c-Jun in *P. monodon* as new potential candidate genes could be further investigated for the development of potential disease resistance markers in the giant tiger shrimp. The transcriptome data produced by the present study could also assist the discovery of SNPs directly associated with desirable traits. Moreover, only 2.13% of the unigenes in this study matched *P. monodon* sequences in the GenBank non-redundant database, while 36,852 new unigenes were discovered—significantly broadening our knowledge of the *P. monodon* transcriptome. The large number of transcripts and molecular markers obtained in this study should provide a strong basis for future further genomic research on shrimp.

## Additional Information and Declarations

### DNA Deposition

The following information was supplied regarding the deposition of DNA sequences: The sequence is available at NCBI: SRP153251.

### Data Availability

The following information was supplied regarding data availability: The data is available in the Supplemental Files and is also available at the NCBI Genbank database under accession number SRP153251.
